# CX3CR1 is required for airway inflammation by promoting T helper cell survival and maintenance in inflamed lung

**DOI:** 10.1186/1479-5876-9-S2-P3

**Published:** 2011-11-23

**Authors:** Nicolas Glaichenhaus, Cyrille Mionnet, Valérie Julia

**Affiliations:** 1 Université de Nice-Sophia Antipolis, Institut National de la Santé et de la Recherche Médicale, Valbonne, France

## Background

Allergic asthma is a T helper type 2 (Th2)-dominated disease of the lung. In people with asthma, a fraction of CD4-positive T cells express the CX3CL1 receptor, CX3CR1, and CX3CL1 expression is increased in airway smooth muscle, lung endothelium and epithelium upon allergen challenge. In this study, we have investigated the role of CX3CR1/CX3CL1 in the development of allergic asthma in mice.

## Results

We found that untreated CX3CR1-deficient mice or wild-type mice treated with CX3CR1-blocking reagents showed reduced lung disease upon allergen sensitization and challenge [1]. Transfer of wild-type CD4-positive T cells into CX3CR1-deficient mice restored the cardinal features of asthma, and CX3CR1-blocking reagents prevented airway inflammation in CX3CR1-deficient recipients injected with wild-type Th2 cells. We found that CX3CR1 signaling promoted Th2 survival in the inflamed lungs, and injection of B cell leukemia/lymphoma-2 protein (BCl-2)-transduced CX3CR1-deficient Th2 cells into CX3CR1-deficient mice restored asthma. CX3CR1-induced survival was also observed for Th1 cells upon airway inflammation but not under homeostatic conditions or upon peripheral inflammation.

## Conclusion

CX3CR1/CX3CL1 play a critical role in the development of allergic asthma in mice making that these molecules attractive therapeutic targets in asthma.
